# Multiple Sclerosis: Therapeutic Strategies on the Horizon

**DOI:** 10.7759/cureus.24895

**Published:** 2022-05-10

**Authors:** Ramya Talanki Manjunatha, Salma Habib, Sai Lahari Sangaraju, Daniela Yepez, Xavier A Grandes

**Affiliations:** 1 Graduate Medical Education, Kempegowda Institute of Medical Sciences, Bangalore, IND; 2 Medicine and Surgery, Institute of Applied Health Science, Chittagong, BGD; 3 Research, PES Institute of Medical Sciences and Research, Kuppam, IND; 4 Faculty of Medicine, Universidad Catolica de Santiago de Guayaquil, Guayaquil, ECU; 5 General Physician, Universidad Catolica Santiago de Guayaquil, Guayaquil, ECU

**Keywords:** risk factors for multiple sclerosis, symptomatic management of multiple sclerosis, pathogenesis of multiple sclerosis, multiple sclerosis exacerbation, disease-modifying therapies for ms, relapsing-remitting multiple sclerosis

## Abstract

Multiple sclerosis (MS) is a chronic disease affecting the brain and the spinal cord. It is a chronic inflammatory demyelinating disease of the central nervous system. It is the leading cause of non-traumatic disability in young adults. The clinical course of the disease is quite variable, ranging from stable chronic disease to rapidly evolving debilitating disease. The pathogenesis of MS is not fully understood. Still, there has been a rapid shift in understanding the immune pathology of MS away from pure T cell-mediated disease to B cells and microglia/astrocytes having a vital role in the pathogenesis of MS. This has helped in the emergence of new therapies for management. Effective treatment of MS requires a multidisciplinary approach to manage acute attacks, prevent relapses and disease progression and treat the disabling symptoms associated with the disease. In this review, we discuss the pathogenesis of MS, management of acute relapses, disease-modifying therapies in MS, new drugs and drugs currently in trial for MS and the symptomatic treatment of MS.

All language search was conducted on Google Scholar, PubMed, MEDLINE, and Embase till February 2022. The following search strings and medical subheadings (MeSH) were used: "Multiple Sclerosis", "Pathogenesis of MS", and "Disease-modifying therapies in MS". We explored literature on the pathogenic mechanisms behind MS, management of acute relapses, disease-modifying therapies in MS and symptomatic management.

## Introduction and background

Multiple sclerosis (MS) is an autoimmune and inflammatory disorder of the central nervous system (CNS). It is characterized by demyelination of the neuronal axons to various degrees and proliferation of glial cells (gliosis) [1-3]. The presentation of MS is variable as it can present with a wide range of symptoms, including sensory, motor, autonomic and visual impairment. It can present as episodes of reversible neurological deficits to neurodegeneration with the recurrence of episodes [1-5]. MS bears a burden globally, and the prevalence has been increasing, with the highest risk of MS being seen in North American, European, and Australian descent (>100 cases per 100,00 population) and the least in countries near the equator [1-4]. Recent studies have estimated the prevalence in the US to be 1 million [6]. MS affects women three times more than men. It mostly primarily affects young people between the ages of 20-40 years. It is one of the major causes of neurological dysfunction in young people [3,5]. The aetiology of MS is not fully understood. It is categorized as a T cell-mediated autoimmune disease with contribution from B cells and microglia [2]. It involves interaction between genetic, metabolic, dietary, and environmental factors, including Vitamin D exposure, Epstein Barr virus (EBV) and smoking [3-5]. MS can be divided into various categories based on the course of the disease (Table 1).

**Table 1:** Categories of MS RR: Relapsing-remitting, MS: Multiple Sclerosis [1,3]

Categories	Short Description
Relapsing-remitting MS (RRMS)	Most common form is characterized by relapses and remissions.
Secondary progressive MS (SPMS)	Seen in RR type of MS where with time the relapses decrease in number but the disease progresses.
Primary progressive MS (PPMS)	Progressive worsening of neurological symptoms from the beginning.
Progressive relapsing MS (PRMS)	Rarest form characterized by gradual aggravation, occasional recurrence and continuous progression between relapses.

The course of the disease varies among patients [1]. Although significant research and progress have been made in treating MS, there is no established cure yet. Currently, the treatment of MS mainly consists of (1) Treating the exacerbations/relapses- the initial step is to rule out precipitants like infections and treat them. Acute management mainly comprises high dose intravenous corticosteroids or plasmapheresis reserved for those not responding to steroids [4,7]; (2) Symptomatic management- patients with MS suffer from various symptoms, including cognitive impairment, pain, fatigue, bladder dysfunction, and spasticity [4]. These symptoms are treated through pharmacological and non-pharmacological methods. Patients need a holistic and multidisciplinary approach to symptomatic management of MS [4,7]; (3) Disease-modifying therapies (DMTs)- These drugs are disease modifiers that reduce the incidence of relapses and delay the progression of the disease [8]. 

There has been rapid progress in DMTs in the previous two decades [4]. Although many drugs are being used to treat MS, there are only 15 drugs approved by the FDA until 2020. The first drug approved for MS was Interferon beta in 1993 [8]. These drugs are immunomodulators that prevent damage associated with inflammation caused by T and B lymphocytes, macrophages, antibodies, complement and microglia [7]. There has been a shift in understanding the pathogenesis of MS, away from T cell-mediated disease to B cells and microglia also having a role in disease causation [3,7]. Although there have been a lot of new drugs, their use has been inhibited by high cost, adverse effects and not enough proven evidence of use in patients [7,8]. Studies have shown that the lifetime direct medical costs of MS are estimated to be $4.8 million, making it the second most expensive healthcare condition, only behind heart failure [9,10]. The US healthcare system has spent $18.8 billion on DMTs for MS in 2018 alone. This high cost has caused difficulty accessing DMTs due to insurance exclusions [10]. DMTs are also associated with a wide range of adverse effects given their mechanism of action on the immune system like infections, bradycardia, heart blocks, macular oedema, infusion reactions, injection-site reactions, and secondary autoimmune adverse effects, such as autoimmune thyroid disease [11]. 

With an increase in the number of new therapeutic drugs in MS and a better understanding of the pathogenesis, the review aims to provide a concise understanding of the pathogenesis of MS, highlight the management of acute relapses, disease-modifying therapies in MS, new drugs and drugs currently in trial for MS and the symptomatic treatment of MS. We also throw light on clinical trials conducted to prove the efficacy of MS drugs and their safety profile.

## Review

Pathogenesis of MS

The pathogenesis of MS is heterogeneous, and it is a combination of genetic factors, environmental factors and inflammatory processes. Following are some of the potential risk factors for MS (Table 2).

**Table 2:** Risk Factors for Multiple Sclerosis EBV: Epstein-Barr virus, HLA: Human leukocyte antigen, IL-RA: Interleukin receptor subunit alpha [12-14]

GENETIC	ENVIRONMENTAL
Female gender	Infection - EBV, Mycoplasma pneumonia
First-degree relatives	Temperate climate
HLA DR15/DQ6, IL2RA, IL7RA alleles	Low levels of Vitamin D
Gene methylation, somatic mutations	Lack of sunlight exposure
Disease-modifying genes	Cigarette smoking
Race - Caucasians, North European descent	Obesity
	Areas away from the equator

The pathology of MS as an autoimmune disorder was described with the help of a single animal model, experimental autoimmune encephalomyelitis (EAE) [15,16]. Inflammatory demyelination was induced by peripheral immunization with myelin protein components such as myelin basic protein (MBP), proteolipid protein (PLP) and myelin oligodendrocyte glycoprotein (MOG) [15]. Based on the model, several immunological pathways in MS were explored. The hallmarks of MS are inflammation, demyelination, remyelination, reactive gliosis, axonal loss and neurodegeneration [17,18]. These can occur focally or diffusely, predominantly affecting the white matter and involving grey matter and deep brainstem nuclei [18,19]. 

**Figure 1:**
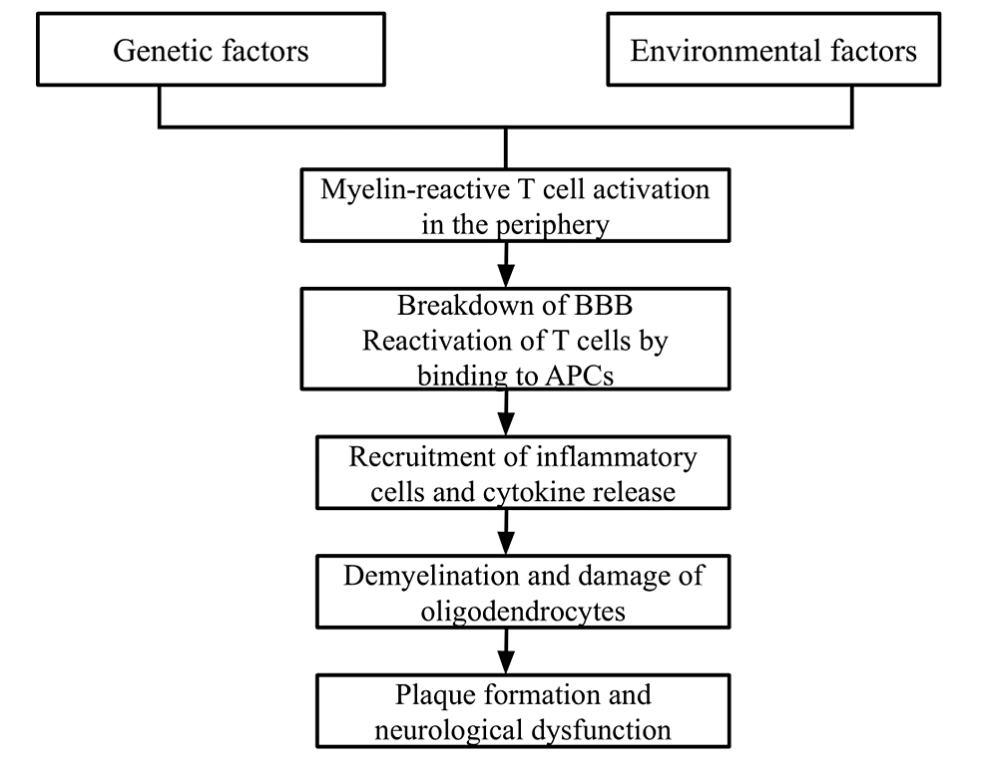
Pathogenesis of Multiple Sclerosis BBB: Blood-brain barrier, APCs: Antigen-presenting cells Image credits: Ramya Talanki Manjunatha

The hallmark of MS pathology is focal plaques of demyelination. The typical plaque has been believed to be located around the central vein [20]. The location of the lesion determines the type of clinical manifestations. Activating myelin-reactive T cells in the periphery is the beginning of the inflammation, leading to the breakdown of the blood-brain barrier (BBB). An interaction mediates the transmigration of leukocytes into the CNS between the integrins, which are expressed on the surface of the leukocytes with their ligand, the cell adhesion molecules (CAMs), which are expressed on the endothelial cells. This process is facilitated by the upregulation and expression of various adhesion molecules, chemokines and metalloproteinases (MMPs) [2,15,21]. After entering the BBB, they are reactivated by presenting a specific antigen to the antigen-presenting cells (APCs). The APCs include B cells, microglia, macrophages, and dendritic cells [16,22]. This triggers an inflammatory response leading to the release of cytokines and chemokines, which bring in inflammatory cells like T cells, B cells, and monocytes and also result in activation of microglia and macrophages, leading to myelin damage [2,22]. A diagram depicting the pathogenesis of MS can be seen below (Figure 1).

Th1 cells are known to mediate the major part of MS pathogenesis [23]. Th1 cells produce interferon-gamma (IFN-γ), tumour necrosis factor-beta (TNF-β), interleukin 2 (IL-2), and lymphotoxin, while Th17 cells are also inflammatory and produce interleukin-17 (IL-17), IL-22, IL-21, and IFN-γ [18,24]. They activate the macrophages and microglia to release cytokines and reactive oxygen species (ROS), which can strip the myelin and destroy oligodendrocytes. CD8+ effector cells can also release IL-17 and IFN-γ, contributing to myelin damage. They also release cytotoxic granules resulting in axonal dissection. IFN-γ augments the disease by increasing the expression of HLA class I and II molecules which enhances the antigen-presenting abilities of macrophages and astrocytes [18]. The mechanism by which IL-17 works is still not clearly understood. But studies on mice who were IL-17 deficient showed a reduction in clinical severity [25]. A study done showed that myelin basic protein (MBP) induced CD4+ cell proliferation and IL-17 production co-related with the number of active plaques found on magnetic resonance imaging (MRI) [26]. On the other hand, CD4+ Th2 cells produce anti-inflammatory cytokines like IL-4, IL-5, IL-6, IL-10, and IL-13. These cytokines limit the Th1 mediated cell injury [2,18]

Although more focus was given to CD4+ cells, it now appears that CD8+ cells also play a role in the pathogenesis of MS [27]. It is shown that the perivascular cuffs of the active demyelinating plaques in MS contain 50 times more CD8+ cells than the CD4+ cells. They also often present in disease-progression-associated cortical plaques [18,28]. CD8+ cells recognize peptides through MHC class I, which are expressed ubiquitously. They produce cytolytic proteins such as perforin and granzymes, which mediate the suppression and inactivation of CD4+ cells. CD8+ cells also kill glial cells exposing the axons. These cells cause transection of axons and increase vascular permeability leading to activation of oligodendrocyte death. They impair the myelin repair process due to oligodendrocyte death [19,23]. Pathogenic CD8+ cells may also contribute to MS pathology by secreting IFN-γ and IL-17. A subset of CD8+ cells defined by the surface expression of CD161 has been reported to be elevated in MS patients [29]. 

Subsets of T cells known as regulatory T cells also play a role in the pathogenesis of MS. Treg cells act by suppressing inflammation, these are deficient in autoimmune diseases, and they function by suppressing the effector CD4+ T cell subsets [30]. The dysregulation of markers that induce autoimmune suppression on the Treg cells has been linked with MS pathogenesis [18]. Lower T-reg suppressive capabilities lead to an increase in pro-inflammatory cytokines like IL-6, IL-17, and IFN-γ. They also cause activation of autoantibody-producing B cells [31]. FoxP3+ Tregs are known as professional suppressor cells, and they exert a dominant inhibition of activation of other cells through cell contact. Presently studies are being done on Tregs as they have been recognized as potential therapeutic targets in MS [32].

Although MS was considered mainly a T cell-mediated disease, recent studies have shown the involvement of B cells in the pathogenesis of MS. Polyclonal antibodies in the cerebrospinal fluid (CSF) of MS patients, known as oligoclonal bands, point to B cells' involvement. These are observed in about 95% of MS patients. Clonally expanded B cells are present in the brain parenchyma, meninges, and CSF of MS patients [18,33]. They are present in increased frequency in the beginning stages of MS. Studies have also shown that increased B cell frequency is related to the faster progression of the disease [34]. B cells are also known to produce pro-inflammatory cytokines like lymphotoxin, TNF-alpha and anti-inflammatory cytokines like IL-10 produced by naive B cells besides producing antibodies [35]. Meningeal inflammation and B cell follicle-like structures adjacent to subpial cortical lesions also point to B cell involvement in MS [2]. These B cell follicles express CD20+. Clinical trials have revealed that anti-CD20 monoclonal antibodies are highly effective in limiting new relapsing disease activity. In addition, these B cells also contribute to pathogenesis by (1) Antigen presentation to T cells and helping autoproliferation of brain homing T cells; (2) They produce soluble toxic factors resulting in oligodendrocyte and neuronal injury; (3) they contribute to the formation of lymphoid aggregates (ectopic) in the meninges; and (4) Provide a reservoir for Epstein-Barr virus (EBV). Hence B cells act as a therapeutic target in MS [36].

Other cells which have been indicated in the pathogenesis of MS are microglia. Microglia are known as resident immune cells of the CNS and are essential for brain homeostasis. They have been increasingly recognized for their role in CNS development and function [37]. In MS, microglia become 'disease-associated microglia' (DAM) by altering their transcription profile. DAM cells display an inflammatory phenotype in the EAE model [38]. Microglia are present throughout all the stages of lesion formation as inflammatory drivers. They are found in slowly expanding lesions that are linked to disease progression. They also are present diffusely in the cortex and contribute to the loss of synapses. Microglia are said to be protective in the early stages of the disease, but as the disease progresses, they become pathogenic later [39]. A study tracing microglia populations in a lysophosphatidylcholine-induced demyelination model has identified that microglia activation limits infiltration of peripheral macrophages and proliferation in the CNS, suggesting microglia are the main drivers of the innate immune system. Emerging therapies have been targeting microglia to treat MS [40]. Several studies are still active in identifying the mechanisms behind the pathogenesis of MS to help find better therapeutic options that will prevent the development of MS. 

Treatment of acute relapses

The relapses in MS are defined as a new or worsening neurological deficit lasting 24 hours or more in the absence of infection or fever [41]. About 80-90% of patients with MS present with a relapsing-remitting course but also are observed in patients with secondary progressive MS with superimposed relapses [42]. The relapses could represent new demyelination or be due to inflammation of a previously existing lesion. Relapses/ exacerbations in MS are one of the biggest concerns to the patients as they cause both functional and emotional impairment to the patient. Moreover, the unpredictability of the exacerbations further impacts the quality of life [43]. It is essential to rule out conditions that may cause 'pseudo-exacerbations' like fever, infections (most common are urinary tract and upper respiratory), stress, and heat exposure [41,44].

The usual course of MS relapse is completed with a repair period leading to remission or sometimes complete recovery. However, residual deficits can persist, which may contribute to the progression of disability [41]. It has been shown that frequent relapses in the first two years and shorter first interval attacks have been predicted to reach disability endpoints in a shorter time [43]. Therefore, accurate management of acute relapses should be a significant concern as it may help shorten and lessen the disability associated with the disease course.

The treatment of MS exacerbations for decades now has involved the use of anti-inflammatory treatments like corticosteroids (CS) and adrenocorticotropic hormone (ACTH). They help accelerate the recovery and lessen the severity of the attack [45]. Corticosteroids are considered the first-line treatment for relapses in MS [46]. CS have shown potent anti-inflammatory and immunosuppressive properties. In total, 90-95% of the drug administered is plasma-bound to mainly corticosteroid-binding globulin and albumin. They are metabolized by the liver and have a half-life of 60-90 minutes [41,43]. CS acts by several mechanisms such as the resolution of brain oedema, reduction of permeability in the blood-brain barrier, redistribution of circulating T cells with a transient decrease in CD4+ cells, reduces the expression of adhesion molecules and HLA class II molecules, reduce immunoglobulin synthesis and increase levels of transforming growth factor-beta in CNS, facilitation of apoptosis of activated immune cells and inhibition of T cell activation, reduces the expression of pro-inflammatory cytokines, reduces the levels of metalloproteinases (MMPs) and increases the levels of tissue inhibitors of MMPs [41-46].

An analysis was done comparing CS or ACTH to placebo in acute relapse management for MS. It compromised six randomized, double-blind controls including 377 patients (199 treatment, 178 placeboes) within eight weeks of relapse onset. The study concluded that treatment with CS or ACTH decreased the risk of worsening or being stable on the expanded disability scale (EDSS) at five weeks from relapse (OR 0.37, 95% CI, 0.24-0.57). It showed a trend of better efficacy with methylprednisolone (MP) and intravenous (IV) treatment but no significant difference between the short (five days) or long (15 days) duration of therapy [47]. The most commonly used CS are methylprednisolone and dexamethasone. A Cochrane meta-analysis of oral vs IV CS, which included five studies, showed no significant difference in efficacy for relapse treatment between the two routes of administration and no difference in MRI imaging [48]. Another recent study which included six trials involving 419 participants, 210 (oral) and 209 (IV) groups, showed no difference in relapse rate at six months between the two groups. However, there was an increase in the adverse effect rate in the oral group compared to the IV group [46].

The usual practice for relapse patients is to use intravenous methylprednisolone 1 g/day for 3-7 days/ ACTH 80-120 units/day intramuscular (IM) or subcutaneous (SC) for one week/ oral prednisone 500-1250 mg/day divided for 3-7 days. A further high dose of up to 2g IV-MP can be used for 3-5 days if there is no clinical response to the first IV-MP treatment after two weeks [45,49]. A point of controversy is the use of oral CS for tapering after pulsed IV-MP. Perumal et al. conducted a retrospective study that did not show any long-term difference between patients who received oral taper vs patients who did not [50]. Hoogervest et al. studied 35 MS patients enrolled in a clinical trial of oral IFN-beta who had acute relapses, treated with long term oral steroids showed short term reductions in brain volume to patients who did not [51]. An animal study conducted by Reder et al. also showed that on termination of IV-MP, relapses followed, which were prevented by oral steroid taper for 14 days [52]. There is no evidence that long-term treatment with CS would delay the long-term progression of disability associated with MS. A systematic review did not reduce the risk of being worse after treatment with IV-MP for a long term (more than six months) [53]. Also, IV-MP has been shown to induce faster recovery in patients with optic neuritis. The North American Optic Neuritis Treatment Trial randomized 457 patients with optic neuritis for three days with 1g IV-MP followed by 14 days of oral prednisone taper. After one month, patients taking IV-MP showed faster recovery and improved visual function, but no change was seen with oral CS [54]. Although corticosteroids are associated with a long list of adverse effects, short-term use for relapses is only associated with mild side effects. However, some patients can exhibit intolerable side effects even with short-term courses of CS. The most commonly encountered include insomnia, depression, irritability, euphoria, dyspepsia, headache, palpitations and acne. Patients with diabetes, cardiac conditions, myasthenia gravis, and patients on warfarin should be monitored while on CS therapy [43,45].

It has been seen that there are patients who do not respond to either CS or ACTH treatment. Alternative therapies have to be considered for these patients like plasmapheresis, IV immunoglobulin, natalizumab, or cyclophosphamide. According to the American Academy of Neurology (AAN), therapeutic plasma exchange (TPE) is considered the second line of treatment for steroid-resistant relapsing cases of MS [55]. TPE is an extracorporeal technique for blood purification performed by separating plasma from blood, exchanging the plasma with donor plasma/albumin solution and returning the other components like red blood cells to the patient. It is also referred to as a blood cleansing procedure. The changes induced by antibody redistribution are considered to be causing beneficial effects in TPE. TPE removes immunoglobulins, cytokines, complement factors, and immune complexes, which are involved in the disease process of MS [56]. A randomized sham-controlled double-blinded study was performed on patients with MS. Those who weren't on any immunosuppressive drugs and failed to recover with IV steroid treatment were treated with TPE. The study showed moderate to greater improvement in neurological disability in eight out of 19 (42.1%) patients in the active treatment group vs one out of 17 (5.9%) in the sham treatment group [57]. Another prospective study on 20 patients who received TPE for 21 steroid-resistant relapses showed marked to moderate improvement in function in 76% of patients with uni or bilateral optic neuritis and 87.5% of patients with relapses other than optic neuritis [58]. Another retrospective study performed in 2017 on a Portuguese cohort of patients with MS relapses included 46 patients on whom corticosteroids were used for a mean of 6.09 days, with 41.30% of patients showing no improvement. Others who gave only mild improvement were given a mean of 7.39 plasmapheresis sessions which showed complete EDSS recovery in 41.30% and partial recovery in 39.13% of patients [59]. 

Mayo Clinic performed a study on 59 patients from Jan 1984 to June 2000 with severe acute attacks of demyelinating disease, which showed clinical improvement in 44.1%. Male patients had preserved reflexes, and those who had early initiation of treatment showed marked improvement [60]. Keegan et al. studied the relation of pathological patterns to TPE treatment. He found four prominent pathological patterns in the early stages of MS. Treatment with TPE showed a marked improvement with pattern II, which shows immunoglobulin deposition and complement activation. Hence, plasmapheresis has shown clinical improvement in patients with severe MS relapse [61]. The number of cycles needs to be decided regarding the patient profile to achieve a better clinical outcome. TPE is associated with a few adverse effects, such as anemia and symptomatic hypotension. Rarely heparin-induced thrombocytopenia and bacteremia due to central infection were noted [59,60]. TPE can also be used in pediatric patients. A retrospective study on pediatric patients treated with apheresis for demyelinating disease between 2007 and 2011 showed improved outcome rates similar to adults [62]. But pediatric apheresis patients are associated with a higher complication rate. A retrospective study of 186 children treated with apheresis between 1994 and 2022 showed adverse effects mainly due to citrate toxicity, volume depletion and the need for vascular access. Therefore, TPE in children requires a multidisciplinary approach [63]. 

Immunoadsorption (IA) is an alternative method to plasmapheresis. IA selectively removes immunoglobulins while preserving other plasma proteins. There are two options available for IA now (1) Single-use tryptophan-based absorbers and (2) Reusable protein A based adsorbers. A randomized control study done between 2016 to 2018 on 61 patients (TPE-30, IA-31) showed more significant improvement in patients on IA than TPE after four weeks. Even though IA showed superiority, there has not been enough evidence on the use of IA in relapses for MS [64]. IA can be considered superior to TPE for pregnant patients as it preserves protective plasma proteins needed in pregnancy rather than discarding them [65].

Another option for acute relapse management is IV immunoglobulin (IVIG). IVIG can act in various mechanisms to help resolve MS pathogenesis. IVIG can neutralize circulating antibodies against myelin proteins found in MS patients. They can also bind to B cell receptors leading to downregulation of antibody production. In addition to it, they also inhibit the phagocytosis of myelin mediated by macrophages. Also, they can modify the balance between Th1 and Th2 cells leading to decreased production of inflammatory cytokines. IVIG attenuates complement activation inhibiting complement-mediated demyelination [66]. There are four randomized, double-blind controls performed on the use of IVIG for acute relapses in MS. They were performed in Poland, Israel, Austria and Denmark. All these studies showed beneficial anti-inflammatory effects of IVIG, reduced relapse rates and improvements in MRI [67-70]. Another study was done to compare the use of IVIG in combination with IV-MP vs MP alone. Only a slighter better remission was seen in the IVIG group; hence there is no evidence to use IVIG as an add-on treatment to IV-MP for better outcomes [71]. Although improvement is shown with IVIG use, no studies have convincingly documented the efficacy of IVIG over other treatments in acute relapse management; a cohort of patients is small and the methods employed are in question [72].

Disease-modifying therapies in MS

Disease-modifying therapies (DMTs) have been considered the mainstay of treatment for MS. By 2019, 17 DMTs were approved for use in MS by the US Food and Drug Administration (FDA). They have various mechanisms of action which aren't fully understood due to a void in completely understanding MS pathogenesis. All the current DMTs are for treating relapsing forms of MS, and ocrelizumab is the only approved drug for primary progressive multiple sclerosis. DMTs are administered either as injectables, orally, or as infusions [73].

Injectables

Interferon-beta (IFN-beta): IFN-beta was the first approved drug for MS treatment in 1993. Although many new drugs have been coming to light, IFN-beta still remains a first-line treatment in the current decision-making process for relapsing-remitting (RR)MS management [74]. IFN-beta is a naturally occurring cytokine in our body with a wide range of anti-inflammatory properties. Recombinant forms of this have been developed: IFN-beta-1a, which is available for IM use (Avonex®) or as SC use (Rebif®); IFN-beta-1b for SC use (Betaseron®) (Extavia®); and Pegylated IFN-beta-1a for SC use (Plegridy®) [75].

IFN-beta 1b is made from bacterial cells (*E.coli*) by replacing one of the three cysteines with serine to maintain the stability of the structure. In addition, bacterial cells cannot glycosylate recombinant protein; hence IFN-beta-1b lacks the carbohydrate group found in natural IFN-beta, whereas IFN-beta-1a are produced from Chinese hamster ovary cells, which have N-terminal methionine needed for glycosylation. Hence IFN-beta-1a is ten times more immunogenic than IFN-beta-1b. Thus a higher dose of IFN-beta-1b is required to produce the same biological effect as IFN-beta-1a [76]. 

The mechanism of action of IFN-beta is a complex process. It acts by decreasing the production of pro-inflammatory cytokines like IL-17 and osteopontin, increasing the production of anti-inflammatory cytokines like IL-10 and IL-4, IFN-beta-1a limits leukocyte migration across BBB, aids in CNS repair and recovery by promoting nerve-growth factor (NGF) expression and increasing production of CD56 natural killer cells [77]. Antibodies developing against IFN-beta are a significant concern as they can weaken the cellular response to IFN-beta and neutralize its therapeutic effect. Hence are known as neutralizing antibodies (NAbs). Sorensen et al. measured NAbs in 541 patients treated with IFN-beta every 12 months for up to 60 months. Patients developed NAbs independent of age, sex, and disease duration, and the relapse rates were higher during antibody-positive periods (0.64-0.70) than during the negative periods (0.43-0.46). Hence, the presence of these antibodies reduces the drug's clinical effect, prompting the use of another drug [78]. Sominada et al. analyzed 1115 MS patients treated with one of the INF-beta preparations from 1-120 months for the presence of NAbs using a protein induction assay. He found that NAb titres from IFN-beta-1a treated patients to be higher than IFN-beta-1b [79]. Although in such cases, IFN-beta needs to be discontinued. However, it is seen that these patients with positive NAbs can turn negative with continued use of IFN-beta [80].

Pegylated interferon is another preparation of IFN, which involves the covalent addition of an inert polyethylene glycol (PEG) molecule to improve pharmacological efficacy. PEG increases the mass of the drug, inhibits proteolysis, and reduces renal elimination, increasing the drug's half-life, hence requiring less dosing, causing lesser side effects [81]. Calabresi et al. conducted a double-blind, randomized phase 3 ADVANCE trial in 26 countries on patients with RRMS receiving SC pegylated IFN-beta-1a 125 microgram once every 2/4 weeks for 48 weeks. Adjusted annual relapse rates were 0.39 in the placebo group vs 0.256 in the every two weeks drug group and 0.288 in the four-week group [82].

The most common adverse effects of IFN-beta use are flu-like symptoms and injection site reactions. The most frequently seen laboratory abnormalities are leukocytopenia and elevation of liver enzymes [83]. An open labelled study was conducted by Logan Clubb et al. on 30 patients with RRMS being treated with IFN-beta reported injection site reactions in 80% such as redness, swelling or itching, 60% showed flu-like symptoms including nausea, fever, chills, malaise, less than 10% reported weight gain, tremor, irritability or weakness. Only four patients discontinued the drug due to adverse effects after four months [84]. 

Glatiramer acetate (GA): Glatiramer acetate (Copaxone®, Glatopa®) is a synthetic copolymer composed of polypeptides formed by four amino acids (glutamic acid, lysine, alanine and tyrosine) mimicking the myelin basic protein (MBP) for SC use [85]. GA acts by modification of immune processes involved in the pathogenesis of MS. The mechanism of actions of GA is the displacement of MBP from the binding site in MHC II complex, T cell receptor antagonism against immunodominant epitope 82-100 of MBP, induction of the shift from Th1 to Th2 response, migration of Th2 type cell through the BBB into CNS, decrease in inflammatory cytokine IFN-gamma and increase in IL-10, IL-4, IL-6, neuroprotection due to increase in the production of brain-derived neurotrophic factor (BDNF) by GA specific T cells and induction of CD4+ CD25+ regulatory T cells which are suppressive [86-89].

GA is effective in reducing the relapse rate in RRMS. Ford et al. conducted a prospective study on RRMS patients using GA for a decade. A total of 232 patients were included in the intention to treat group (who received at least one dose of GA), of which 124 withdrew from the study. After ten years of treatment, the relapse reduction rate was <80% [90]. Comi et al. conducted a double-blind, randomized placebo-controlled study in patients with RRMS receiving GA versus placebo to measure MRI disease activity. Two-hundred and ninety patients were randomized to receive 20 mg GA or placebo by daily SC injection. Treatment with GA after nine months showed a significant reduction in enhancing lesions on MRI compared to the placebo (-10.8, 95% confidence interval -18.0 to -3.7, p = 0.003) [91]. Studies were later done to see the efficacy of higher doses with lesser dosage frequency in treating RRMS. Khan et al. conducted a randomized, double-blind study in 1524 RRMS patients across 142 sites; 1404 received 40 mg SC thrice-weekly vs placebo for 12 months (GALA study group). GA 40 mg was associated with a 34% reduction in risk of confirmed relapses compared to placebo (annual relapse rate = 0.331 vs 0.505, p<0.0001). They also noted a significant decrease in T1 (44.8%) and T2 (34.7%) lesions on MRI. They were also safe and well-tolerated; therefore, GA 40 mg thrice a week is safe and also provides the convenience of fewer SC injections [92].

Cohen et al. conducted another randomised control trial (RCT) to compare the two dosages of GA. A difference was noted between the two dosage groups as early as three months, favouring the 40 mg group [93]. GA has a favourable side effect profile overall. The most common AE is local injection site reactions which reduce over time. Approximately 10% of patients develop post-injection symptoms like flushing, chest tightness, dyspnea, and urticaria. They have also been shown to develop lipoatrophy and skin necrosis [94]. GA can be used as an alternative therapy for patients who do not tolerate IFN-beta due to persistent side effects and for patients who develop NAbs against IFN-beta [95]. Johnson et al. conducted a study to assess the long term safety and efficacy of GA, which showed GA is well tolerated and safe for long term use. It also showed that delaying therapy with GA increases the risk of neurological disability; hence GA should be used in the early course of RRMS [95,96].

Ofatumumab: Ofatumumab (Kesimpta®) is a recombinant human anti-CD20 monoclonal immunoglobulin antibody approved for use in relapsing forms of MS in 2020. It can be used in relapsing forms, including clinically isolated syndrome (CIS), RRMS and secondary progressive (SP)MS. It is the first self-injectable subcutaneous B cell therapy administered at home. The recommended dose is 20 mg SC at weeks 0, 1, and 2, followed by 20 mg once monthly starting at the 4th week [97]. Ofatumumab is an anti-CD20 antibody. It effectively induces complement-dependent toxicity and also mediates antibody-dependent cellular cytolysis. It binds to a region distinct from other anti-CD20 antibodies, including the smaller and larger loop of CD20 receptors [98]. Sorensen et al. conducted a phase 2 RCT on 38 patients who received ofatumumab infusions (100 mg, 300 mg or 700 mg) or placebo two weeks apart. In the 24th week, they received alternate treatment. Ofatumumab showed significant depletion of B cells (measured by CD 19 status). Also, new lesions on MRI were suppressed by >99% in the first 24 weeks after starting treatment. No serious adverse effects were noted; the most common one was an adverse reaction after the first dose [99]. A phase 2b double-blind RCT study (MIRROR study) to assess the dose-response effects of ofatumumab showed a reduction in new lesions by 65% in those receiving the drug vs placebo (p<0.001). B cell depletion was noted; also the study showed that complete depletion of B cells was not necessary for successful treatment [100].

The approval of the ofatumumab for treating MS was based on the ASCLEPIOS I and II phase 3 studies. The study was a double-blind RCT conducted at multiple sites for 30 months. The median trial time was 1.6 years. Patients with relapsing MS were randomly assigned to receive SC ofatumumab (946 patients received 20 mg every four weeks after 20 mg loading doses on days 1, 7, and 14) or oral teriflunomide (936 patients received 14 mg daily). The annualized relapse rate (ARR) were 0.11 vs 0.22 in trial 1 and 0.10 vs 0.25 in trial 2 (ofatumumab vs teriflunomide respectively). The percentage of disability worsening at six months was 8.1% with ofatumumab vs 12% with teriflunomide (hazard ratio,1.35; CI, 0.95 to 1.92; p=0.09) [101]. Similarly, a greater reduction in MRI lesions and lowering of neurofilament light chain, a marker of neuroaxonal damage, was seen with ofatumumab. But the change in brain volume did not vary between the two treatments. The most common side effect was injection-related reactions (20.2% in ofatumumab vs 15% in the teriflunomide group). Other adverse events in at least 10% of patients were nasopharyngitis, headache, upper respiratory tract infections and urinary tract infections in those treated with ofatumumab. Serious adverse effects like serious infections and neoplasms were reported in 9.1% of patients treated with ofatumumab vs 7.9% with teriflunomide [101]. Larger and long term trials are required to determine the long term efficacy and safety of ofatumumab compared to other DMTs.

Oral DMTs

Teriflunomide: Teriflunomide (Aubagio®) is an oral DMT that the US FDA approved in 2012 to treat MS's relapsing forms. The recommended dose is 7 mg or 14 mg orally once daily [102]. Teriflunomide is a reversible, selective inhibitor of dihydroorotate dehydrogenase (DHODH), a rate-limiting mitochondrial enzyme involved in the de-novo pyrimidine synthesis [103]. Blocking pyrimidine synthesis interrupts the cell cycle in the S phase, decreasing the proliferation of T and B cells and limiting their involvement in the pathogenesis of MS. It only inhibits activated rapidly proliferating lymphocytes and doesn't affect resting lymphocytes [104]. An experimental study conducted by Yilmaz et al. on mice showed that B6 mice treated with teriflunomide had reduced memory B cells, plasma cells and plasmablast ratios [105]. Teriflunomide acts by inhibition of DHODH, which inhibits B and T cell proliferation, inhibition of T cell receptors causing interference with integrin function, prevents interaction of T cells with APCs causing the decreased formation of the immunological synapse and decreases the release of pro-inflammatory cytokines from activated monocytes in a DHODH dependent manner [106,107]. In a clinical study TERIDYNAMIC, it was observed that teriflunomide causes selective changes in the T cell subset and T cell receptors in RRMS patients. They used an antigen-specific set-up to show that teriflunomide caused the suppression of high-affinity T cells. T cells from RRMS patients exhibited higher levels of oxidative phosphorylation and glycolysis than the healthy controls [108].

The clinical efficacy of teriflunomide has been studied extensively in relapsing forms of MS. Miller et al. conducted the Teriflunomide Multiple Sclerosis (TEMSO) trial, which is a double-blind, randomized controlled phase 3 trial conducted in 1088 RMS patients who received 7 mg or 14 mg once daily teriflunomide vs placebo for 108 weeks. ARR was the primary endpoint; it was significantly reduced in both the 7 mg group (0.370%, p=0.0002) and the 14 mg group (0.369%,p=0.005). There was also a reduction in disability progression, total lesion volume, and gadolinium-enhancing lesions on T1 among both groups compared to the placebo [109]. An extension study was conducted by O'Connor et al. to report safety and efficacy outcomes for up to nine years after the TEMSO trial. ARRs improved compared to the core trial:0.198 and 0.215 for patients continuing teriflunomide 7 mg and 14 mg, respectively. The safety profile was similar, with no new adverse effects, EDSS remained stable and sustained disability remained low [110]. Miller et al. conducted another double-blind RCT phase 3 trial (TOPIC) to assess the efficacy and safety of teriflunomide in patients with a first clinical episode suggestive of MS. Patients with the clinically isolated syndrome (CIS) across 20 countries were randomly assigned in a 1:1:1 ratio to once-daily (OD) teriflunomide 14 mg, 7 mg or placebo for 108 weeks. Patients treated with teriflunomide significantly reduced the risk of relapse, teriflunomide 14 mg (hazard ratio {HR} 0.574, 95% CI, p=0.0087) and 7 mg (HR 0.628, p=0.0271) vs the placebo. Teriflunomide-treated patients also had a reduction in the number of new MRI lesions. Adverse events in the teriflunomide group at least 2% greater than in the placebo group were an increase in alanine aminotransferase (ALT), hair thinning, diarrhoea, paraesthesia, and upper respiratory tract infection [111]. TENERE, a rater blinded phase 3 RCT, compared the efficacy of teriflunomide with IFN-beta-1a. The study showed that teriflunomide was as effective as IFN-beta-1a in ARR with no unexpected adverse effects [112]. Hence teriflunomide remains an effective option for managing relapsing forms of MS with long-term benefits. The findings of these studies can be noted below (Table 3).

**Table 3:** Clinical trials to study the efficacy of teriflunomide in relapsing forms of MS RCT: Randomized control trial, ARR: Absolute risk reduction, IFN-beta-1a: Interferon beta-1 alpha, HR: Hazard ratio, ALT: Alanine amino-transferase, OD: once daily, Gde: Gadolinium-enhanced [109-112] Miller at al. 2012 [109]; O' Connor et al. 2016 [110]; Miller et al. 2012 [111]; Varmersch et al. 2014  [112]

Clinical Trial	Study Design	Study Participants	Clinical Outcomes	MRI Outcomes	Adverse Effects
TEMSO Miller at al. 2012	Multicenter placebo-controlled double-blind phase III RCT	1088 subjects with MS assigned to 7 mg, 14 mg or placebo for 108 weeks	Lower ARR with teriflunomide ( 0.37) vs placebo(0.54)	Both teriflunomide doses were superior to placebo in reducing MRI lesions.	More common with teriflunomide- diarrhoea, nausea, hair thinning, elevated liver enzymes
TEMSO extension O' Connor et al. 2016	Multicenter double-blind phase III RCT	742 patients with RRMS assigned to OD 7mg or 14mg teriflunomide, 9-year long study	ARR improved as compared to core study. ARR=0.198 &0.215 (7&14 mg)	Gde lesions reduced in patients who switched from placebo to teriflunomide	Same as the original study
TOPIC Miller et al. 2014	Randomised double-blind, placebo-controlled phase III trial	618 patients with CIS assigned to OD 14mg or placebo for 108 weeks	Teriflunomide significantly reduced the risk of relapse vs the placebo (14mg (HR 0.574,95% CI, p=0.0087) and 7mg (HR 0.628, p=0.0271))	Teriflunomide reduced the risk of a new MRI lesion vs placebo	Adverse events occurred in atleast 10% of teriflunomide groups and with an incidence 2% higher than placebo were increased ALT, diarrhoea, paraesthesia
TENERE Varmersch et al. 2014	Phase 3 rater-blinded RCT	324 patient with relapsing MS assigned to OD teriflunomide 7 or 14mg or SC IFN-beta-1a 44 microgram	ARR significantly higher with the 7 mg teriflunomide group. No difference was noted in ARR between 14mg or IFN-beta-1a.		Safety profile consistent with previous studies

Dimethyl fumarate (DMF): Dimethyl fumarate (Tecfidera®) is an oral DMT, a fumaric acid ester approved by the US FDA in 2013 for use in relapsing forms of MS in the adult population. It is given as a 120 mg capsule by mouth twice daily for one week, followed by 240 mg twice daily after that [113]. The mechanism by which DMF exerts its therapeutic effect is not yet established. It has been shown that DMF acts by activation of the Nrf2 transcriptional pathway, which is involved in modulating responses at the cellular level to oxidative stress. DMF also upregulates the expression of various detoxifying and antioxidant response proteins. DMF, along with its active metabolite, monomethyl fumarate (MMF), have also been shown to increase the recycling of glutathione which reduces reactive oxygen species (ROS) in astrocytes oligodendrocytes and hippocampal cells [114]. DMF and MMF increase redox potential, glutathione levels, and mitochondrial membrane potential in a concentration-dependent manner. They result in an improvement in cell viability. Hence DMF and MMF are cytoprotective for neurons against cellular injury induced by oxidative stress and loss via the up-regulation of an Nrf2 dependent antioxidant response [115]. DMF is also shown to inhibit microglial and astrocytic inflammation by decreasing the synthesis of pro-inflammatory mediators like nitric oxide, TNF-alpha, IL-1 beta and IL-6. It was also associated with decreased extracellular signal-regulated kinases (ERK) phosphorylation [116]. Nrf2 independent mechanisms like activation of G coupled protein receptor hydroxy-carboxylic acid receptor (HCA2) are also involved in therapeutic anti-inflammatory effects. MMF is also known to act as a nicotinic receptor agonist [117].

The efficacy of DMF was evaluated in many clinical trials. DEFINE, a placebo-controlled phase 3 study, was conducted to determine the effectiveness of DMF in RRMS patients. Patients were randomly assigned to receive oral DMF at a dose of 240 mg twice daily (BID), DMG at 240 mg thrice daily (TID) or placebo. For two years. The primary endpoint was the number of patients who had relapsed by two years, which was significantly low in the two DMF groups compared to placebo (27% with DMF BID, 26% with DMF TID, 46% with placebo, p<0.001). There was also a decrease in ARR, rate of disability progression and number of MRI lesions with DMF treatment vs placebo. Adverse events noted were flushing and gastrointestinal (GI) events like diarrhoea, nausea, upper abdominal pain, decreased lymphocyte counts and increased ALT levels [118]. Fox et al. conducted a similar trial of the Comparator and oral fumarate in RRMS (CONFIRM) with glatiramer acetate as a reference comparator, concluding that DMF and GA reduced relapse rates and improved relapse rates radiological outcomes as compared to placebo. There was no difference noted in the twice-daily vs thrice-daily dosing of DMF [119]. A subsequent extension of the CONFIRM and DEFINE study was the ENDORSE. In ENDORSE patients were randomized to receive 240 mg of DMF BID or TID as a continued dosage from previous trials; those who received a placebo now were given either DMF 240 mg BID or TID. The ARR at the end of five years was 0.138 for the BID group. DMF treatment was associated with continuously low clinical and MRI disease activity in RRMS patients [120]. The most common adverse effects of DMF are flushing and gastrointestinal events. A few cases of progressive multifocal leukoencephalopathy (PML) were noted in patients on DMF, one of whom died. Providing patients with information on treating adverse effects before starting treatment will help patients tolerate the drug better [121].

Diroximel fumarate (DRF) (Vumerity®) is a compound similar to DMF with the same active metabolite MMF and a better gastrointestinal side effect profile. It is an oral fumarate approved in the USA for relapsing forms of MS. It is administered as a 231 mg capsule by mouth twice daily for one week, followed by two 231 mg capsules taken twice daily after that [122]. Naismith et al. conducted a double randomized control phase 3 EVOLVE-MS-2 trial to compare the GI tolerability of DRF vs DMF over five weeks in patients with RRMS. Either DRF or DMF was administered to patients and two self-administered GI symptom scales. DRF treated patients reported a significant reduction (46%) in the number of days with GI symptom intensity score >/= 2 compared to patients treated with DMF (rate ratio:0.54, p=0.0003) [123]. Patients on DRF showed lower rates of GI adverse effects like diarrhoea, nausea, vomiting, and abdominal pain than those on DMF (34.8% vs 49.0%). Also fewer patients discontinued DRF as compared to DMF (1.6% vs 5.6%) [123].

Sphingosine 1-Phosphate Receptor Modulators

Fingolimod: Fingolimod (Gilenya®) was the first sphingosine 1-phosphate (S1P) receptor (S1PR) modulator and also the first oral DMT to be approved by the FDA for use in relapsing forms of MS. The recommended dose is 0.5 mg capsule OD. S1P is a signalling molecule generated through the metabolism of the cell membrane sphingolipid through phosphorylation of sphingosine by sphingosine kinase 1 or 2. S1P is involved in the embryonic development of CNS and cardiovascular systems. SP1 interacts with five G protein-coupled receptors (GPCRs:S1PR-S5PR) [124].

T cells, B cells and natural killer (NK) cells mainly express S1PR1 and others to a smaller extent except for S1P2R. It antagonizes S1PR1, which is required for lymphocyte egress from the lymph nodes resulting in a significant reduction in circulating lymphocytes and inflammatory cell infiltration of the CNS [125]. Fingolimod also has been shown to decrease the production of the Th17 subset of cells producing IL-17 which plays a major role in the pathogenesis of MS [126]. Miron et al. conducted experimental studies on fetal neuronal cells to study the action of fingolimod (FTY720) on oligodendrocyte progenitor cells (OPCs). The study revealed that FTY720 induces time-dependent modulation of S1PR on human OPCs needed for the remyelination process [127]. Fingolimod is also shown to mediate ERK phosphorylation in astrocytes via activation of S1P1R [128]. A clinical phase 4 trial was conducted to study the impact of fingolimod on NK cells, which showed an increased frequency of circulating NK cells (CD56, CD94 mature NK cells) which might limit the anti-tumour NK cell activity in fingolimod treated patients [129].

Two large multicenter, double-blind RCT phase 3 studies were conducted to show the efficacy of fingolimod in treating relapsing forms of MS. FREEDOM trial is a double-blinded RCT conducted over 24 months of 1272 patients with RRMS who had not received IFN-beta or GA for at least three months and natalizumab in the past six months. The subjects received fingolimod at doses of 0.5 mg, 1.25 mg or placebo. At the end of two years the ARR was 0.18 in 0.5 mg group, 0.16 in 1.25 mg group, and 0.40 with placebo (p<0.001) [130]. Subjects on fingolimod also showed a reduced risk of disability progression and gadolinium-enhanced lesions on MRI. There was significant preservation of brain volume loss in subjects on 0.5 mg fingolimod vs placebo (-0.84 vs -1.31%). Subjects receiving fingolimod discontinued the drug due to adverse events like bradycardia, atrioventricular block, macular oedema, elevated liver enzymes and mild hypertension [130]. The FREEDOM II trial was later conducted to assess the efficacy and safety of fingolimod on RRMS patients. The study reconfirmed the results obtained by the FREEDOM I trial [131]. Cohen et al. conducted another phase 3 double-blinded RCT, TRANSFORMS, to compare the efficacy of fingolimod in RRMS patients. Subjects received either fingolimod 1.25 mg or 0.5 mg orally or IM IFN-beta-1a at a weekly dose of 30 mcg. At the end of 12 months, the ARR was significantly lower in both groups receiving fingolimod (-0.20 in 1.25 mg group, 0.16 in 0.5 mg group) vs IFN-beta group (0.33). A similar pattern was noted with MRI outcomes. No significant differences were seen regarding the progression of disability. The 1.25 mg group showed two fatal infections (disseminated primary varicella-zoster and herpes simplex encephalitis) [132]. An extension of the TRANSFORMS study demonstrated continued efficacy of long-term fingolimod with a consistent safety profile as the core trial in maintaining the lower rate of disease activity and improved efficacy after switching from IFN-beta-1a to fingolimod [133].

Lublin et al. conducted a phase 3 double-blind placebo-controlled RCT to determine the efficacy of oral fingolimod in primary progressive MS conducted over five years, which concluded that oral fingolimod did not slow disease progression in primary progressive (PP)MS however, the safety results were consistent with previous studies [134]. Derfuss et al. recently conducted a cross-sectional study ACROSS to study the long term efficacy of fingolimod use. One hundred and seventy-five patients with RRMS participated in the study, 104 were classified as high exposure and the other as low exposure. At the end of ten years, disability progression was lower in the high exposure group vs low exposure group (34.7% vs 56.1%) [135].

A major problem with the prescription of fingolimod is the first-dose monitoring requirements before starting the drug. But recent changes to the development of in-home fingolimod first-dosing processes are helping clinicians meet these challenges [136]. Another critical risk factor that should be considered when stopping fingolimod treatment is the rebound effect. The effects were mostly seen 12 weeks after discontinuation of treatment, and this can be due to changes in lymphocyte phenotypes that can promote disease activity. The rebound is more severe than a typical MS relapse; hence care should be taken while withdrawing the drug [124,137].

Siponimod: Siponimod (Mayzent®) is a highly selective S1PR1 (on astrocytes) and S1PR5 (on oligodendrocytes) modulator. Due to structural modification, it doesn't act at S1PR3, potentially minimizing adverse effects and resulting in a shorter half-life. It also causes a long-lasting internalization of S1P receptors upon binding [138]. Like fingolimod, siponimod acts by reducing lymphocyte egress. It influences both T and B cells with a pronounced effect on CD4 T cells. It is also shown to decrease oligodendrocyte and axonal loss, concluding that it can protect axons from demyelination [139].

A phase II trial (BOLD) in RRMS showed a reduction in new T2 lesions and gadolinium-enhancing lesions. Given its therapeutic effect on MRI lesions and tolerability, a phase 3 trial was warranted [140]. Kappos et al. conducted a placebo-controlled large double-blind phase III RCT (EXPAND) of siponimod for patients with secondary progressive MS across 31 countries. Subjects were either given 2 mg OD siponimod or placebo for three years or until the occurrence of a prespecified number of confirmed disability progression (CDP) events. The primary outcome was time to three months of confirmed disability progression measured by EDSS. Siponimod significantly reduced the risk of three month CDP by 21% compared to placebo (p=0.013) [141]. It also showed beneficial effects on ARR, T2 lesion volume, and gadolinium-enhancing MRI lesions. Also, a benefit in percent brain volume change was noted (p=0.0002 vs placebo). Adverse effects were reported more in the siponimod group vs placebo (89 vs 82%). Adverse events included lymphopenia, increased ALT, bradycardia and bradyarrhythmia at treatment initiation, macular oedema, hypertension and convulsions. The frequency of infections, malignancies and death did not vary between the groups [141]. A secondary analysis performed on the EXPAND study provided class II evidence that for patients with SPMS, siponimod had a significant benefit on cognitive processing speed [142]. Cao et al. performed a meta-analysis of RCTs that evaluated siponimod; based on the findings; they were uncertain whether siponimod was beneficial for MS patients as there was no high-certainty data to establish its use. Hence, more new studies with better methodology, longer follow-ups, and comparisons with other DMTs are needed [143].

Ozanimod: Ozanimod is an agonist of S1PR1 and S1PR5 with 27 fold selectivity for S1PR1. It induces durable S1P1 receptor internalization and degradation, resulting in a rapidly reversible reduction in circulating lymphocytes. It also has been shown to reduce lymphocyte subsets that express cytokine receptor 7. It has high oral bioavailability, a short half-life and effectively crosses BBB. Unlike fingolimod, it doesn't act on S1PR3 receptors present on the myocytes and hence has a better safety profile [144]. Ozanimod also exhibits a neuroprotective effect. Studies on a mouse model (EAE) revealed that ozanimod could dampen EAE glutaminergic synaptic alterations by increasing the local inflammatory response mediated by activated microglia and T cells resulting in decreased clinical disability [145]. 

The approval by the FDA of ozanimod was based on two phase III trials. The RADIANCE phase 3 study was conducted to confirm the safety and efficacy of ozanimod vs IFN-beta-1a in individuals with relapsing forms of MS. The study was a 24 month, double-blinded, double-dummy multicentre study. Patients were randomly assigned daily oral ozanimod 1.0 or 0.5 mg or weekly IM IFN-beta-1a 30 microgram. At the end of 24 months, patients with ozanimod had a significantly lower rate of clinical relapses when compared to IFN-beta-1a (0.17 with 1 mg, 0.22 with 0.5 mg and 0.28 with IFN-beta-1a). Adverse events were more in the IFN-beta group than in the ozanimod, leading to treatment discontinuation. No bradycardia or atrioventricular block cases were noted with ozanimod use [146]. SUNBEAM was a similar phase 3 trial which included 1346 patients with RMS across 152 sites in 20 countries for 12 months. Similar results were seen with patients on ozanimod demonstrating a significantly lower relapse rate than IFN-beta-1a. The incidence of malignancy was similar to IFN-beta; half of the malignancies reported were non-melanoma skin cancers [147]. An open-label phase III trial (DAYBREAK) was conducted recently with 2495 participants who completed one of the four parent trials. The study concluded that continuing or switching to ozanimod 1 mg was associated with a low ARR, was generally well-tolerated, and no new safety concerns were raised [148]. A post hoc analysis of phase 3 trials was conducted to show plasma neurofilament light chain concentration (pNFL) as a biomarker for neuroaxonal damage and disease activity. The study concluded that pNFL was associated with RMS's clinical and radiological measures of disease and treatment effects, supporting its use as a biomarker. Ozanimod was associated with decreased levels of pNFL vs IFN-beta [149]. Therefore, ozanimod has an excellent advantage in reducing ARR and MRI lesions due to damage reduction and repair enforcement.

Ponesimod: Ponesimod (Ponvory®) is a selective and rapidly reversible S1P1 modulator. It acts as a functional antagonist with potential immunomodulating activity. It acts similar to other S1P modulators by reducing the amount of circulating peripheral lymphocytes. A study in healthy subjects showed an above 80% decrease in lymphocyte count, which was re-established within seven days of stopping treatment, hence higher safety and faster return of anti-inflammatory activity. Adverse effects reported with ponesimod were anxiety, cough, dyspnea, influenza, insomnia, peripheral oedema, and increased ALT [150].

A phase IIb double-blind placebo-controlled RCT studied the efficacy and safety of ponesimod in RRMS patients. OD treatment with 10, 20 or 40 mg ponesimod significantly reduced the number of new T1 lesions, and ARR was lower than placebo [151]. OPTIMUM was then conducted, the first phase 3 study comparing two oral DMTs for two oral DMTs for relapsing MS. The study was a multicenter double-blind, active-comparator RCT conducted in 1133 patients with RRMS. Patients were randomized to receive 20 mg ponesimod or 14 mg teriflunomide once daily and the placebo for 108 weeks, with a 14-day gradual titration of ponesimod starting at 2 mg to prevent the first dose cardiac effects of ponesimod and a follow-up period of 30 days. At the end of 108 weeks, a 30% reduction of ARR was noted in the ponesimod group compared to teriflunomide (0.202 vs 0.290) [152]. Ponesimod was also superior to teriflunomide on MRI activity, brain volume loss, and fatigue and showed no evidence of disease activity status but not confirmed disability accumulation. Incidences of adverse events were similar in both groups. The FDA approval of this drug in 2021 for relapsing forms of MS in adults was after the OPTIMUM study [152]. 

Cladribine: Cladribine (Mavenclad®) is the first short course DMT used in MS. The FDA approved it in 2019 for relapsing forms of MS, RRMS and active secondary progressive disease in adults. It is an oral medication given once per year for two years. Each course has two cycles, 4-5 days long and one month apart. The dose is estimated to be 10 to 20 mg daily for an adult of average body weight for 4-5 days [153]. Cladribine is a synthetic purine nucleoside analogue (prodrug) phosphorylated to its active form by deoxycytidine kinase (DCK). Cladribine triphosphate, its active form, accumulates in the cell resulting in disruption of cellular metabolism, DNA damage and apoptosis. It targets T cells, B cells and dendritic cells due to their high DCK content [154]. It modifies the disease course of MS by causing depletion of autoreactive T and B cells involved in demyelination, axonal degeneration and neuronal loss [155].

In phase 3 placebo-controlled RCT (CLARITY) conducted by Giovannoni et al., 1326 patients with RRMS were randomly assigned to receive one of two cumulative doses of cladribine tablets (3.5 mg or 5.25 mg/kg based on body weight) or placebo given in two/four short courses for first 48 weeks, then in two short courses at week 48 and 52. At the end of 96 weeks, patients who received cladribine had significantly lower ARR vs the placebo (0.14 and 0.15 vs 0.33,p<0.001), lower risk of three-month sustained progression of disability and lower brain lesion count on MRI [156]. Adverse events were more in the cladribine group, with the most common one being lymphocytopenia (21.6%, 31.5% vs 1.8%). Other adverse events noted were leukopenia, alopecia, upper respiratory tract infection and uterine leiomyoma [156]. A two-year extension study of the CLARITY trial demonstrated no improvement in efficacy from continuing cladribine after the first two courses of treatment [157].

Oral cladribine in Early MS (ORACLE MS) was another double-blind RCT of oral cladribine, which demonstrated a higher risk reduction in the cladribine group vs placebo for time to conversion to clinically definite MS. The trial was terminated in between due to the development of severe lymphopenia in a few subjects [158]. Comi et al. analyzed the long-term lymphocyte count changes in pooled data from CLARITY, CLARITY extension and PREMIERE registry. He concluded that although reductions in T and B cells were noted, recovery began soon after treatment in years 1 and 2. Hence cladribine is a form of immune reconstitution therapy [159]. Given the adverse effect profile of cladribine, it is to be used only in patients not responding to other DMTs or tolerating other DMTs. The ONWARD study, a phase 2 study, was conducted to evaluate the safety and efficacy of cladribine in patients still experiencing active MS relapse despite treatment with IFN-beta. The study provided Class I evidence that cladribine added to IFN-beta reduced relapses and MRI lesion activity and increased the incidence of lymphopenia [160]. During the CLARITY trial, data showed economic benefits with cladribine as it used fewer healthcare resources [161].

Intravenous Infusion Treatments

Mitoxantrone: Mitoxantrone (Novantrone®) is a synthetic anthracenedione derivative that is an antineoplastic and immunomodulator. Mitoxantrone acts by macrophage-mediated suppression of B cell, T helper and T cytotoxic lymphocyte function, inhibition of antigen-induced lymphocyte proliferation, inhibition of myelin degradation in a dose-dependent manner, disrupts DNA synthesis and repair, impairs secretion of IFN-gamma, TNF-alpha and IL-2 and induction of apoptosis of B lymphocytes [162,163].

Hartung et al. conducted a multicentre placebo-controlled, double-blind RCT (MIMS) to evaluate the safety and efficacy of mitoxantrone 5mg/m(2) and 12 mg/m(2) every three months intravenously in RRMS and SPMS patients for 24 months. At the end of 24 months, a significant (<0.0001) difference was noted in the primary endpoint for the 12 mg/m(2) group compared to the placebo (difference 0.30 {95% CI 0.17-0.44}). Mitoxantrone 12 mg/m(2) was generally well tolerated and reduced the progression of the disease and clinical exacerbations [164]. This led to the approval of mitoxantrone by the FDA in 2000 for reducing neurological disability or the frequency of relapse in patients with secondary progressive, progressive relapsing or worsening relapsing-remitting MS patients [164]. Mitoxantrone has also been used as first-line treatment in patients new to any DMT with an aggressive RR course, which showed improved EDSS and MRI activity. Despite potential adverse events, it may be considered an option in selected patients with very active early MS [165].

A double-blind clinical trial of mitoxantrone vs IV-MP demonstrated a significant improvement in EDSS after one year of treatment (p<0.0022). The total number of relapses, mean number of relapses and a total number of lesions on MRI scan were significantly decreased in the mitoxantrone group vs IV-MP. Hence it has a role in treating patients with frequent exacerbations and rapid disease progression [166]. Mitoxantrone use has been associated with a lot of well known adverse effects. The short-term adverse effects most frequently seen during all trials include nausea & vomiting, alopecia, an increase in the risk of infections, and an increase in liver enzymes and bilirubin. It may be associated with amenorrhea, infertility, or persistent amenorrhea in the long term. The most serious risks are cardiotoxicity and leukaemia. Cardiotoxicity increases with a higher cumulative dose, limiting the maximum dose to 140 mg/m(2). Therapy-related acute leukaemia (TRAL), especially acute leukaemia, is another major concern with the use of mitoxantrone, limiting its use [167].

Natalizumab and Alemtuzumab; Natalizumab (Tysabri®) is the first human monoclonal antibody indicated in treating RRMS. Although it was efficient in treating active RRMS, its use is limited due to a serious adverse event related to its use (progressive multifocal leukoencephalopathy). With the advent of new and safer therapeutic agents, its use has been limited to serving as second-line therapy for MS [168].

Alemtuzumab (Lemtrada®) is a humanized anti-CD52 monoclonal antibody approved by FDA in 2014 for use in RRMS patients. Due to the drug's safety profile, the FDA recommends the use of lemtrada only in people who have an inadequate response to two or more MS therapies [169].

Ocrelizumab: Ocrelizumab is an intravenously administered humanized anti-CD 20 monoclonal antibody approved by the FDA in 2017 for use in relapsing forms of MS in adults. It selectively depletes CD 20 expressing B cells through ADCC, antibody-dependent cellular phagocytosis, complement-dependent cytotoxicity and apoptosis. Ocrelizumab preserves humoral immunity due to plasma cells, and innate and adaptive immunity [170].

The approval of ocrelizumab in RRMS was based on two identical phase 3 trials (OPERA I and II), where subjects were randomly assigned to receive IV ocrelizumab at a dose of 600 mg every four weeks or SC IFN-beta-1a at a dose of 44 micrograms three times weekly for 96 weeks. At the end of the trial, the ARR was low in the ocrelizumab group in both trials (46% in study 1 and 47 % in study 2, p<0.0001). Infusion-related reactions were the most common adverse event noted [171]. It is the first drug approved for use in primary progressive MS. In a phase 3 RCT (ORATORIO) with 732 patients with PPMS, subjects were given either 600 mg ocrelizumab or placebo every 24 weeks for at least 120 weeks. The percentage of patients with 12 week and 24 week confirmed disability progression was lower in the ocrelizumab group vs placebo (32.9% vs 39.3% at 12 weeks, 26.9 vs 35.7). It was also associated with lower rates of MRI progression vs placebo. Additional comparative and long term benefit-risk data will be useful to clearly define the place of ocrelizumab in MS management [172].

Symptomatic management in MS

Symptom management is an essential component of MS patient care. Although many disease-modifying therapies are being found for MS treatment, they can only slow the progression of the disease or reduce disease severity. Still, symptomatic management of MS is the key to improving the quality of life and daily functioning of patients. MS needs holistic management as symptoms can be due to motor dysfunction, cranial nerve symptoms, autonomic nervous system dysfunction, psychiatric problems, and pain symptoms. Patients should be treated functionally, emotionally, vocationally, and socially. Many of these symptoms are underrecognized and overlooked, causing hindrance to the recognition of first-line and approved treatments for these symptoms. Many of the symptoms are treated by off-label use medications [173-174]. Currently, many neuromodulation techniques are being studied to address different neurological symptoms. However, they aren't being used on a large scale. Some methods include intrathecal baclofen pump, deep brain stimulation, bladder stimulation, spinal cord stimulation and transcranial magnetic stimulation [175]. These techniques can improve functional disability, which constitutes a major burden in MS patients. A table depicting the symptoms associated with MS and its management can be seen below (Table 4).

**Table 4:** Management of symptoms in MS patients FDA: Food and drug administration, PT: Physical therapy, OD: Once daily, PFT: Pelvic floor therapy, CS: Corticosteroids, TMS: Transcranial magnetic stimulation, SCS: Spinal cord stimulation, CBT: cognitive behavioural therapy, TENS: Transcutaneous electrical nerve stimulation, TCAs: Tricyclic antidepressants [173-177]

Symptoms	Pharmacological Treatment	Non-Pharmacological Treatment
Impaired gait, balance and co-ordination	Dalfampridine-FDA approved potassium channel blocker for improved walking (10 mg OD orally), meclizine, promethazine	Mobility aids, fall prevention stratergies, physiotherapy (PT), gait training
Tremors and ataxia	High doses of isoniazid, carbamazepine, propranolol, glutethmifr, topiramate	PT, rehabilitation, stereotactic operations - deep brain stimulation thalamotomy of ventralis intermedius
Spasticity	Baclofen (oral and intrathecal), tizanidine, dantrolene, cannabis, botulinum toxin, intrathecal CS	PT, hydrotherapy, TMS
Fatigue	Amantadine, modafinil, methylphenidate, 4-aminopyridine, pemoline	Energy conservative measures, mobility assistance, cooling therapy, aerobic training
Bladder disturbance	Anticholinergics to reduce detrusor activity, alpha-blockers, desmopressin, oxybutynin, botulinum toxin A	Bladder training, pelvic floor therapy (PFT), electrical stimulation, TMS, SCS
Bowel dysfunction	Metoclopramide, domperidone for bowel motility, laxatives like lactulose and bisacodyl	PFT, PT, sufficient fluid and fibre intake, enema
Sexual dysfunction	Men - Sildenafil, Tadalafil, intracavernous vasodilator agents like alprostadil Women - flibanserin	Counseling, lubricants, sexual aids to enhance stimulation
Cognitive dysfunction	Acetylcholinesterase inhibitors, memantine, beta interferons	Cognitive rehabilitation, retraining programme for specific attention deficits
Depression	Antidepressants	Counselling, psychotherapy - cognitive behavioural therapy (CBT)
Visual symptoms	Gabapentin or memantine in pendular nystagmus, baclofen for upbeat nystagmus	Adaptive equipment, environmental modifications
Pain	TCAs, anti-convulsants like gabapentin	Counseling, yoga and meditation, acupuncture, PT, transcutaneous electrical nerve stimulation (TENS), SCS
Trigeminal neuralgia	Carbamazepine, baclofen,gabapentin	CBT, radiofrequency rhizotomyrhizotomy
Mood disorders	Antidepressants	CBT, exercise, yoga and meditation

## Conclusions

Management of MS has come a long way with the introduction of DMTs helping reduce relapses, disease progression, and accumulation of lesions on MRI. But there is still a gap in treating progressive forms of MS, and hence more research is necessary for managing patients of this category. Although a wide range of DMTs therapies has been approved for use in relapsing forms of MS, there are very few guidelines to aid clinicians in selecting DMTs. Selecting DMTs for a patient is a complex process, and has to be selected based on age, category, stage, associated morbid conditions, safety profile, efficacy, and tolerability of the drug.

Treatment of MS patients poses a financial burden globally; hence, cheaper and safe drugs need to be introduced. Ublitixumab is one such drug that is a glycoengineered antibody under FDA review after the ULTIMATE trials recently, which has superior potency relative to current anti-CD 20 antibody therapies, allowing for lower doses, also has shorter infusion times and would be available at an affordable cost with lesser adverse effects. Pathogenesis of MS is another field that is not completely understood but has undergone a lot of progress. Many studies have been running to identify the genes involved in the disease causation. Researchers have been developing medications that have the potential for remyelination and neuroprotection. Hematopoietic stem cell transplant is another promising new therapy on which many clinical trials are being conducted. Although research on DMTs has increased a lot in the previous decade, neuromodulation in symptom management is being overlooked. Therefore, more research is needed in this field as studies have shown efficacy with their use. Finally, effective management of MS needs a multidisciplinary approach to address symptoms, treat acute relapses and reduce long-term disability through disease modification. 

## References

[1] Goldenberg MM (2012). Multiple sclerosis review. P T.

[2] Garg N, Smith TW (2015). An update on immunopathogenesis, diagnosis, and treatment of multiple sclerosis. Brain Behav.

[3] Hauser SL, Cree BA (2020). Treatment of multiple sclerosis: a review. Am J Med.

[4] Doshi A, Chataway J (2017). Multiple sclerosis, a treatable disease. Clin Med (Lond).

[5] Dobson R, Giovannoni G (2019). Multiple sclerosis - a review. Eur J Neurol.

[6] Howard J, Trevick S, Younger DS (2016). Epidemiology of multiple sclerosis. Neurol Clin.

[7] Hart FM, Bainbridge J (2016). Current and emerging treatment of multiple sclerosis. Am J Manag Care.

[8] Wei W, Ma D, Li L, Zhang L (2021). Progress in the application of drugs for the treatment of multiple sclerosis. Front Pharmacol.

[9] Owens GM, Olvey EL, Skrepnek GH, Pill MW (2013). Perspectives for managed care organizations on the burden of multiple sclerosis and the cost-benefits of disease-modifying therapies. J Manag Care Pharm.

[10] Hartung DM (2021). Health economics of disease-modifying therapy for multiple sclerosis in the United States. Ther Adv Neurol Disord.

[11] McGinley MP, Goldschmidt CH, Rae-Grant AD (2021). Diagnosis and treatment of multiple sclerosis: a review. JAMA.

[12] Leray E, Moreau T, Fromont A, Edan G (2016). Epidemiology of multiple sclerosis. Rev Neurol (Paris).

[13] Zarghami A, Li Y, Claflin SB, van der Mei I, Taylor BV (2021). Role of environmental factors in multiple sclerosis. Expert Rev Neurother.

[14] Belbasis L, Bellou V, Evangelou E, Ioannidis JP, Tzoulaki I (2015). Environmental risk factors and multiple sclerosis: an umbrella review of systematic reviews and meta-analyses. Lancet Neurol.

[15] Bar-Or A, Oliveira EM, Anderson DE, Hafler DA (1999). Molecular pathogenesis of multiple sclerosis. J Neuroimmunol.

[16] Loma I, Heyman R (2011). Multiple sclerosis: pathogenesis and treatment. Curr Neuropharmacol.

[17] Kamm CP, Uitdehaag BM, Polman CH (2014). Multiple sclerosis: current knowledge and future outlook. Eur Neurol.

[18] Baecher-Allan C, Kaskow BJ, Weiner HL (2018). Multiple sclerosis: mechanisms and immunotherapy. Neuron.

[19] Ghasemi N, Razavi S, Nikzad E (2017). Multiple sclerosis: pathogenesis, symptoms, diagnoses and cell-based therapy. Cell J.

[20] Meltzer EI, Costello FE, Frohman EM, Frohman TC (2018). New ways of "seeing" the mechanistic heterogeneity of multiple sclerosis plaque pathogenesis. J Neuroophthalmol.

[21] Ciccarelli O, Barkhof F, Bodini B (2014). Pathogenesis of multiple sclerosis: insights from molecular and metabolic imaging. Lancet Neurol.

[22] Chastain EM, Duncan DS, Rodgers JM, Miller SD (2011). The role of antigen presenting cells in multiple sclerosis. Biochim Biophys Acta.

[23] Guo MF, Ji N, Ma CG (2008). Immunologic pathogenesis of multiple sclerosis. Neurosci Bull.

[24] Fletcher JM, Lalor SJ, Sweeney CM, Tubridy N, Mills KH (2010). T cells in multiple sclerosis and experimental autoimmune encephalomyelitis. Clin Exp Immunol.

[25] Komiyama Y, Nakae S, Matsuki T (2006). IL-17 plays an important role in the development of experimental autoimmune encephalomyelitis. J Immunol.

[26] Hedegaard CJ, Krakauer M, Bendtzen K, Lund H, Sellebjerg F, Nielsen CH (2008). T helper cell type 1 (Th1), Th2 and Th17 responses to myelin basic protein and disease activity in multiple sclerosis. Immunology.

[27] Huseby ES, Huseby PG, Shah S, Smith R, Stadinski BD (2012). Pathogenic CD8 T cells in multiple sclerosis and its experimental models. Front Immunol.

[28] Hauser SL, Bhan AK, Gilles F, Kemp M, Kerr C, Weiner HL (1986). Immunohistochemical analysis of the cellular infiltrate in multiple sclerosis lesions. Ann Neurol.

[29] Annibali V, Ristori G, Angelini DF (2011). CD161(high)CD8+T cells bear pathogenetic potential in multiple sclerosis. Brain.

[30] Zozulya AL, Wiendl H (2008). The role of regulatory T cells in multiple sclerosis. Nat Clin Pract Neurol.

[31] Danikowski KM, Jayaraman S, Prabhakar BS (2017). Regulatory T cells in multiple sclerosis and myasthenia gravis. J Neuroinflammation.

[32] Schmidt A, Oberle N, Krammer PH (2012). Molecular mechanisms of treg-mediated T cell suppression. Front Immunol.

[33] Schirmer L, Srivastava R, Hemmer B (2014). To look for a needle in a haystack: the search for autoantibodies in multiple sclerosis. Mult Scler.

[34] Cepok S, Jacobsen M, Schock S (2001). Patterns of cerebrospinal fluid pathology correlate with disease progression in multiple sclerosis. Brain.

[35] Cencioni MT, Mattoscio M, Magliozzi R, Bar-Or A, Muraro PA (2021). B cells in multiple sclerosis - from targeted depletion to immune reconstitution therapies. Nat Rev Neurol.

[36] Comi G, Bar-Or A, Lassmann H (2021). Role of B cells in multiple sclerosis and related disorders. Ann Neurol.

[37] Walsh AD, Nguyen LT, Binder MD (2021). miRNAs in microglia: important players in multiple sclerosis pathology. ASN Neuro.

[38] Voet S, Prinz M, van Loo G (2019). Microglia in central nervous system inflammation and multiple sclerosis pathology. Trends Mol Med.

[39] Guerrero BL, Sicotte NL (2020). Microglia in multiple sclerosis: friend or foe?. Front Immunol.

[40] Plemel JR, Stratton JA, Michaels NJ (2020). Microglia response following acute demyelination is heterogeneous and limits infiltrating macrophage dispersion. Sci Adv.

[41] Berkovich R (2013). Treatment of acute relapses in multiple sclerosis. Neurotherapeutics.

[42] Myhr KM, Mellgren SI (2009). Corticosteroids in the treatment of multiple sclerosis. Acta Neurol Scand Suppl.

[43] Smets I, Van Deun L, Bohyn C (2017). Corticosteroids in the management of acute multiple sclerosis exacerbations. Acta Neurol Belg.

[44] Anlar O (2009). Treatment of multiple sclerosis. CNS Neurol Disord Drug Targets.

[45] Frohman EM, Shah A, Eggenberger E, Metz L, Zivadinov R, Stüve O (2007). Corticosteroids for multiple sclerosis: I. application for treating exacerbations. Neurotherapeutics.

[46] Lattanzi S, Cagnetti C, Danni M, Provinciali L, Silvestrini M (2017). Oral and intravenous steroids for multiple sclerosis relapse: a systematic review and meta-analysis. J Neurol.

[47] Filippini G, Brusaferri F, Sibley WA, Citterio A, Ciucci G, Midgard R, Candelise L (2000). Corticosteroids or ACTH for acute exacerbations in multiple sclerosis. Cochrane Database Syst Rev.

[48] Burton JM, O'Connor PW, Hohol M, Beyene J (2012). Oral versus intravenous steroids for treatment of relapses in multiple sclerosis. Cochrane Database Syst Rev.

[49] Yamout B, Sahraian M, Bohlega S (2020). Consensus recommendations for the diagnosis and treatment of multiple sclerosis: 2019 revisions to the MENACTRIMS guidelines. Mult Scler Relat Disord.

[50] Perumal JS, Caon C, Hreha S, Zabad R, Tselis A, Lisak R, Khan O (2008). Oral prednisone taper following intravenous steroids fails to improve disability or recovery from relapses in multiple sclerosis. Eur J Neurol.

[51] Hoogervorst EL, Polman CH, Barkhof F (2002). Cerebral volume changes in multiple sclerosis patients treated with high-dose intravenous methylprednisolone. Mult Scler.

[52] Reder AT, Thapar M, Jensen MA (1994). A reduction in serum glucocorticoids provokes experimental allergic encephalomyelitis: implications for treatment of inflammatory brain disease. Neurology.

[53] Ciccone A, Beretta S, Brusaferri F, Galea I, Protti A, Spreafico C (2008). Corticosteroids for the long-term treatment in multiple sclerosis. Cochrane Database Syst Rev.

[54] Beck RW, Cleary PA, Anderson MM Jr (1992). A randomized, controlled trial of corticosteroids in the treatment of acute optic neuritis. The Optic Neuritis Study Group. N Engl J Med.

[55] Cortese I, Chaudhry V, So YT, Cantor F, Cornblath DR, Rae-Grant A (2011). Evidence-based guideline update: plasmapheresis in neurologic disorders: report of the Therapeutics and Technology Assessment Subcommittee of the American Academy of Neurology. Neurology.

[56] Rolfes L, Pfeuffer S, Ruck T (2019). Therapeutic apheresis in acute relapsing multiple sclerosis: current evidence and unmet needs-a systematic review. J Clin Med.

[57] Weinshenker BG, O'Brien PC, Petterson TM (1999). A randomized trial of plasma exchange in acute central nervous system inflammatory demyelinating disease. Ann Neurol.

[58] Trebst C, Reising A, Kielstein JT, Hafer C, Stangel M (2009). Plasma exchange therapy in steroid-unresponsive relapses in patients with multiple sclerosis. Blood Purif.

[59] Correia I, Ribeiro JJ, Isidoro L (2018). Plasma exchange in severe acute relapses of multiple sclerosis - results from a Portuguese cohort. Mult Scler Relat Disord.

[60] Keegan M, Pineda AA, McClelland RL, Darby CH, Rodriguez M, Weinshenker BG (2002). Plasma exchange for severe attacks of CNS demyelination: predictors of response. Neurology.

[61] Keegan M, König F, McClelland R (2005). Relation between humoral pathological changes in multiple sclerosis and response to therapeutic plasma exchange. Lancet.

[62] Koziolek M, Mühlhausen J, Friede T (2013). Therapeutic apheresis in pediatric patients with acute CNS inflammatory demyelinating disease. Blood Purif.

[63] Michon B, Moghrabi A, Winikoff R (2007). Complications of apheresis in children. Transfusion.

[64] Dorst J, Fangerau T, Taranu D (2019). Safety and efficacy of immunoadsorption versus plasma exchange in steroid-refractory relapse of multiple sclerosis and clinically isolated syndrome: a randomised, parallel-group, controlled trial. EClinicalMedicine.

[65] Hoffmann F, Kraft A, Heigl F (2018). Tryptophan immunoadsorption during pregnancy and breastfeeding in patients with acute relapse of multiple sclerosis and neuromyelitis optica. Ther Adv Neurol Disord.

[66] Sorensen PS (2003). The role of intravenous immunoglobulin in the treatment of multiple sclerosis. J Neurol Sci.

[67] Sorensen PS, Wanscher B, Jensen CV (1998). Intravenous immunoglobulin G reduces MRI activity in relapsing multiple sclerosis. Neurology.

[68] Fazekas F, Deisenhammer F, Strasser-Fuchs S, Nahler G, Mamoli B (1997). Randomised placebo-controlled trial of monthly intravenous immunoglobulin therapy in relapsing-remitting multiple sclerosis. Austrian Immunoglobulin in Multiple Sclerosis Study Group. Lancet.

[69] Achiron A, Gabbay U, Gilad R (1998). Intravenous immunoglobulin treatment in multiple sclerosis. Effect on relapses. Neurology.

[70] Lewańska M, Siger-Zajdel M, Selmaj K (2002). No difference in efficacy of two different doses of intravenous immunoglobulins in MS: clinical and MRI assessment. Eur J Neurol.

[71] Sorensen PS, Haas J, Sellebjerg F, Olsson T, Ravnborg M (2004). IV immunoglobulins as add-on treatment to methylprednisolone for acute relapses in MS. Neurology.

[72] Goodin DS, Frohman EM, Garmany GP Jr (2002). Disease modifying therapies in multiple sclerosis: report of the Therapeutics and Technology Assessment Subcommittee of the American Academy of Neurology and the MS Council for Clinical Practice Guidelines. Neurology.

[73] De Angelis F, John NA, Brownlee WJ (2018). Disease-modifying therapies for multiple sclerosis. BMJ.

[74] Filipi M, Jack S (2020). Interferons in the treatment of multiple sclerosis: a clinical efficacy, safety, and tolerability update. Int J MS Care.

[75] Revel M (2003). Interferon-beta in the treatment of relapsing-remitting multiple sclerosis. Pharmacol Ther.

[76] Runkel L, Meier W, Pepinsky RB (1998). Structural and functional differences between glycosylated and non-glycosylated forms of human interferon-beta (IFN-beta). Pharm Res.

[77] Kieseier BC (2011). The mechanism of action of interferon-β in relapsing multiple sclerosis. CNS Drugs.

[78] Sorensen PS, Ross C, Clemmesen KM (2003). Clinical importance of neutralising antibodies against interferon beta in patients with relapsing-remitting multiple sclerosis. Lancet.

[79] Sominanda A, Rot U, Suoniemi M, Deisenhammer F, Hillert J, Fogdell-Hahn A (2007). Interferon beta preparations for the treatment of multiple sclerosis patients differ in neutralizing antibody seroprevalence and immunogenicity. Mult Scler.

[80] Sorensen PS, Koch-Henriksen N, Ross C, Clemmesen KM, Bendtzen K; Danish Multiple Sclerosis Study Group (2005). Appearance and disappearance of neutralizing antibodies during interferon-beta therapy. Neurology.

[81] Kolb-Mäurer A, Sunderkötter C, Kukowski B, Meuth SG (2019). An update on peginterferon beta-1a management in multiple sclerosis: results from an interdisciplinary Board of German and Austrian Neurologists and Dermatologists. BMC Neurol.

[82] Calabresi PA, Kieseier BC, Arnold DL (2014). Pegylated interferon β-1a for relapsing-remitting multiple sclerosis (ADVANCE): a randomised, phase 3, double-blind study. Lancet Neurol.

[83] Bayas A, Rieckmann P (2000). Managing the adverse effects of interferon-beta therapy in multiple sclerosis. Drug Saf.

[84] Logan-Clubb L, Stacy M (1995). An open-labelled assessment of adverse effects associated with interferon 1-beta in the treatment of multiple sclerosis. J Neurosci Nurs.

[85] Arnon R (1996). The development of Cop 1 (Copaxone®), an innovative drug for the treatment of multiple sclerosis: personal reflections. Immunol Lett.

[86] Aharoni R, Teitelbaum D, Arnon R, Sela M (1999). Copolymer 1 acts against the immunodominant epitope 82-100 of myelin basic protein by T cell receptor antagonism in addition to major histocompatibility complex blocking. Proc Natl Acad Sci U S A.

[87] Aharoni R, Teitelbaum D, Leitner O, Meshorer A, Sela M, Arnon R (2000). Specific Th2 cells accumulate in the central nervous system of mice protected against experimental autoimmune encephalomyelitis by copolymer 1. Proc Natl Acad Sci U S A.

[88] Chen M, Valenzuela RM, Dhib-Jalbut S (2003). Glatiramer acetate-reactive T cells produce brain-derived neurotrophic factor. J Neurol Sci.

[89] Hong J, Li N, Zhang X, Zheng B, Zhang JZ (2005). Induction of CD4+CD25+ regulatory T cells by copolymer-I through activation of transcription factor Foxp3. Proc Natl Acad Sci U S A.

[90] Ford CC, Johnson KP, Lisak RP, Panitch HS, Shifronis G, Wolinsky JS (2006). A prospective open-label study of glatiramer acetate: over a decade of continuous use in multiple sclerosis patients. Mult Scler.

[91] Comi G, Filippi M, Wolinsky JS (2001). European/Canadian multicenter, double-blind, randomized, placebo-controlled study of the effects of glatiramer acetate on magnetic resonance imaging--measured disease activity and burden in patients with relapsing multiple sclerosis. European/Canadian Glatiramer Acetate Study Group. Ann Neurol.

[92] Khan O, Rieckmann P, Boyko A, Selmaj K, Zivadinov R (2013). Three times weekly glatiramer acetate in relapsing-remitting multiple sclerosis. Ann Neurol.

[93] Cohen JA, Rovaris M, Goodman AD, Ladkani D, Wynn D, Filippi M (2007). Randomized, double-blind, dose-comparison study of glatiramer acetate in relapsing-remitting MS. Neurology.

[94] Mezzapesa DM, Rovaris M, Filippi M (2005). Glatiramer acetate in multiple sclerosis. Expert Rev Neurother.

[95] Ruggieri M, Avolio C, Livrea P, Trojano M (2007). Glatiramer acetate in multiple sclerosis: a review. CNS Drug Rev.

[96] Johnson KP, Brooks BR, Ford CC (2003). Glatiramer acetate (Copaxone): comparison of continuous versus delayed therapy in a six-year organized multiple sclerosis trial. Mult Scler.

[97] (2020). Novartis Pharmaceuticals Corporation. KESIMPTA® (ofatumumab) injection, for subcutaneous use: US prescribing information. https://www.hcp.novartis.com/products/kesimpta/rms.

[98] Lin TS (2010). Ofatumumab: a novel monoclonal anti-CD20 antibody. Pharmgenomics Pers Med.

[99] Sorensen PS, Lisby S, Grove R (2014). Safety and efficacy of ofatumumab in relapsing-remitting multiple sclerosis: a phase 2 study. Neurology.

[100] Bar-Or A, Grove RA, Austin DJ (2018). Subcutaneous ofatumumab in patients with relapsing-remitting multiple sclerosis: the MIRROR study. Neurology.

[101] Hauser SL, Bar-Or A, Cohen JA (2020). Ofatumumab versus teriflunomide in multiple sclerosis. N Engl J Med.

[102] Cada DJ, Demaris K, Levien TL, Baker DE (2013). Teriflunomide. Hosp Pharm.

[103] Miller AE (2021). An updated review of teriflunomide's use in multiple sclerosis. Neurodegener Dis Manag.

[104] Bar-Or A, Pachner A, Menguy-Vacheron F, Kaplan J, Wiendl H (2014). Teriflunomide and its mechanism of action in multiple sclerosis. Drugs.

[105] Yilmaz V, Ulusoy C, Hajtovic S (2021). Effects of teriflunomide on B cell subsets in MuSK-induced experimental autoimmune myasthenia gravis and multiple sclerosis. Immunol Invest.

[106] Zeyda M, Poglitsch M, Geyeregger R (2005). Disruption of the interaction of T cells with antigen-presenting cells by the active leflunomide metabolite teriflunomide: involvement of impaired integrin activation and immunologic synapse formation. Arthritis Rheum.

[107] Li L, Liu J, Delohery T, Zhang D, Arendt C, Jones C (2013). The effects of teriflunomide on lymphocyte subpopulations in human peripheral blood mononuclear cells in vitro. J Neuroimmunol.

[108] Klotz L, Eschborn M, Lindner M (2019). Teriflunomide treatment for multiple sclerosis modulates T cell mitochondrial respiration with affinity-dependent effects. Sci Transl Med.

[109] Miller AE, O'Connor P, Wolinsky JS (2012). Pre-specified subgroup analyses of a placebo-controlled phase III trial (TEMSO) of oral teriflunomide in relapsing multiple sclerosis. Mult Scler.

[110] O'Connor P, Comi G, Freedman MS (2016). Long-term safety and efficacy of teriflunomide: nine-year follow-up of the randomized TEMSO study. Neurology.

[111] Miller AE, Wolinsky JS, Kappos L (2014). Oral teriflunomide for patients with a first clinical episode suggestive of multiple sclerosis (TOPIC): a randomised, double-blind, placebo-controlled, phase 3 trial. Lancet Neurol.

[112] Vermersch P, Czlonkowska A, Grimaldi LM (2014). Teriflunomide versus subcutaneous interferon beta-1a in patients with relapsing multiple sclerosis: a randomised, controlled phase 3 trial. Mult Scler.

[113] Bomprezzi R (2015). Dimethyl fumarate in the treatment of relapsing-remitting multiple sclerosis: an overview. Ther Adv Neurol Disord.

[114] Deeks ED (2016). Dimethyl fumarate: a review in relapsing-remitting MS. Drugs.

[115] Scannevin RH, Chollate S, Jung MY (2012). Fumarates promote cytoprotection of central nervous system cells against oxidative stress via the nuclear factor (erythroid-derived 2)-like 2 pathway. J Pharmacol Exp Ther.

[116] Wilms H, Sievers J, Rickert U, Rostami-Yazdi M, Mrowietz U, Lucius R (2010). Dimethylfumarate inhibits microglial and astrocytic inflammation by suppressing the synthesis of nitric oxide, IL-1beta, TNF-alpha and IL-6 in an in-vitro model of brain inflammation. J Neuroinflammation.

[117] Dubrall D, Pflock R, Kosinska J (2021). Do dimethyl fumarate and nicotinic acid elicit common, potentially HCA2 -mediated adverse reactions? A combined epidemiological-experimental approach. Br J Clin Pharmacol.

[118] Gold R, Kappos L, Arnold DL (2012). Placebo-controlled phase 3 study of oral BG-12 for relapsing multiple sclerosis. N Engl J Med.

[119] Fox RJ, Miller DH, Phillips JT (2012). Placebo-controlled phase 3 study of oral BG-12 or glatiramer in multiple sclerosis. N Engl J Med.

[120] Gold R, Arnold DL, Bar-Or A (2017). Long-term effects of delayed-release dimethyl fumarate in multiple sclerosis: Interim analysis of ENDORSE, a randomized extension study. Mult Scler.

[121] Blair HA (2019). Dimethyl fumarate: a review in relapsing-remitting MS. Drugs.

[122] Paik J (2021). Diroximel fumarate in relapsing forms of multiple sclerosis: a profile of its use. CNS Drugs.

[123] Naismith RT, Wundes A, Ziemssen T (2020). Diroximel fumarate demonstrates an improved gastrointestinal tolerability profile compared with dimethyl fumarate in patients with relapsing-remitting multiple sclerosis: results from the randomized, double-blind, phase III EVOLVE-MS-2 study. CNS Drugs.

[124] Roy R, Alotaibi AA, Freedman MS (2021). Sphingosine 1-Phosphate Receptor Modulators for Multiple Sclerosis. CNS Drugs.

[125] Chaudhry BZ, Cohen JA, Conway DS (2017). Sphingosine 1-phosphate receptor modulators for the treatment of multiple sclerosis. Neurotherapeutics.

[126] Ward MD, Jones DE, Goldman MD (2014). Overview and safety of fingolimod hydrochloride use in patients with multiple sclerosis. Expert Opin Drug Saf.

[127] Miron VE, Jung CG, Kim HJ, Kennedy TE, Soliven B, Antel JP (2008). FTY720 modulates human oligodendrocyte progenitor process extension and survival. Ann Neurol.

[128] Osinde M, Mullershausen F, Dev KK (2007). Phosphorylated FTY720 stimulates ERK phosphorylation in astrocytes via S1P receptors. Neuropharmacology.

[129] Schwichtenberg SC, Wisgalla A, Schroeder-Castagno M (2021). Fingolimod therapy in multiple sclerosis leads to the enrichment of a subpopulation of aged NK cells. Neurotherapeutics.

[130] Kappos L, Radue EW, O'Connor P (2010). A placebo-controlled trial of oral fingolimod in relapsing multiple sclerosis. N Engl J Med.

[131] Calabresi PA, Radue EW, Goodin D (2014). Safety and efficacy of fingolimod in patients with relapsing-remitting multiple sclerosis (FREEDOMS II): a double-blind, randomised, placebo-controlled, phase 3 trial. Lancet Neurol.

[132] Cohen JA, Barkhof F, Comi G (2010). Oral fingolimod or intramuscular interferon for relapsing multiple sclerosis. N Engl J Med.

[133] Cohen JA, Khatri B, Barkhof F (2016). Long-term (up to 4.5 years) treatment with fingolimod in multiple sclerosis: results from the extension of the randomised TRANSFORMS study. J Neurol Neurosurg Psychiatry.

[134] Lublin F, Miller DH, Freedman MS (2016). Oral fingolimod in primary progressive multiple sclerosis (INFORMS): a phase 3, randomised, double-blind, placebo-controlled trial. Lancet.

[135] Derfuss T, Sastre-Garriga J, Montalban X (2020). The ACROSS study: long-term efficacy of fingolimod in patients with relapsing-remitting multiple sclerosis. Mult Scler J Exp Transl Clin.

[136] Khatri BO (2016). Fingolimod in the treatment of relapsing-remitting multiple sclerosis: long-term experience and an update on the clinical evidence. Ther Adv Neurol Disord.

[137] Chun J, Kihara Y, Jonnalagadda D, Blaho VA (2019). Fingolimod: lessons learned and new opportunities for treating multiple sclerosis and other disorders. Annu Rev Pharmacol Toxicol.

[138] Scott LJ (2020). Siponimod: a review in secondary progressive multiple sclerosis. CNS Drugs.

[139] Tong J, Zou Q, Chen Y (2021). Efficacy and acceptability of the S1P receptor in the treatment of multiple sclerosis: a meta-analysis. Neurol Sci.

[140] Selmaj K, Li DK, Hartung HP (2013). Siponimod for patients with relapsing-remitting multiple sclerosis (BOLD): an adaptive, dose-ranging, randomised, phase 2 study. Lancet Neurol.

[141] Kappos L, Bar-Or A, Cree BA (2018). Siponimod versus placebo in secondary progressive multiple sclerosis (EXPAND): a double-blind, randomised, phase 3 study. Lancet.

[142] Benedict RH, Tomic D, Cree BA (2021). Siponimod and cognition in secondary progressive multiple sclerosis: EXPAND secondary analyses. Neurology.

[143] Cao L, Lao Y, Yao L (2020). Siponimod for multiple sclerosis. Cochrane Database Syst Rev.

[144] Scott FL, Clemons B, Brooks J (2016). Ozanimod (RPC1063) is a potent sphingosine-1-phosphate receptor-1 (S1P1 ) and receptor-5 (S1P5 ) agonist with autoimmune disease-modifying activity. Br J Pharmacol.

[145] Musella A, Gentile A, Guadalupi L (2020). Central modulation of selective sphingosine-1-phosphate receptor 1 ameliorates experimental multiple sclerosis. Cells.

[146] Cohen JA, Comi G, Selmaj KW (2019). Safety and efficacy of ozanimod versus interferon beta-1a in relapsing multiple sclerosis (RADIANCE): a multicentre, randomised, 24-month, phase 3 trial. Lancet Neurol.

[147] Comi G, Kappos L, Selmaj KW (2019). Safety and efficacy of ozanimod versus interferon beta-1a in relapsing multiple sclerosis (SUNBEAM): a multicentre, randomised, minimum 12-month, phase 3 trial. Lancet Neurol.

[148] Fronza M, Lorefice L, Frau J, Cocco E (2021). An overview of the efficacy and safety of ozanimod for the treatment of relapsing multiple sclerosis. Drug Des Devel Ther.

[149] Harris S, Comi G, Cree BA (2021). Plasma neurofilament light chain concentrations as a biomarker of clinical and radiologic outcomes in relapsing multiple sclerosis: post hoc analysis of phase 3 ozanimod trials. Eur J Neurol.

[150] Baldin E, Lugaresi A (2020). Ponesimod for the treatment of relapsing multiple sclerosis. Expert Opin Pharmacother.

[151] Olsson T, Boster A, Fernández Ó (2014). Oral ponesimod in relapsing-remitting multiple sclerosis: a randomised phase II trial. J Neurol Neurosurg Psychiatry.

[152] Kappos L, Fox RJ, Burcklen M (2021). Ponesimod compared with teriflunomide in patients with relapsing multiple sclerosis in the active-comparator phase 3 OPTIMUM study: a randomized clinical trial. JAMA Neurol.

[153] Rammohan K, Coyle PK, Sylvester E, Galazka A, Dangond F, Grosso M, Leist TP (2020). The development of cladribine tablets for the treatment of multiple sclerosis: a comprehensive review. Drugs.

[154] Giovannoni G (2017). Cladribine to treat relapsing forms of multiple sclerosis. Neurotherapeutics.

[155] Jacobs BM, Ammoscato F, Giovannoni G, Baker D, Schmierer K (2018). Cladribine: mechanisms and mysteries in multiple sclerosis. J Neurol Neurosurg Psychiatry.

[156] Giovannoni G, Comi G, Cook S (2010). A placebo-controlled trial of oral cladribine for relapsing multiple sclerosis. N Engl J Med.

[157] Giovannoni G, Soelberg Sorensen P, Cook S (2018). Safety and efficacy of cladribine tablets in patients with relapsing-remitting multiple sclerosis: results from the randomized extension trial of the CLARITY study. Mult Scler.

[158] Leist TP, Comi G, Cree BA (2014). Effect of oral cladribine on time to conversion to clinically definite multiple sclerosis in patients with a first demyelinating event (ORACLE MS): a phase 3 randomised trial. Lancet Neurol.

[159] Comi G, Cook S, Giovannoni G (2019). Effect of cladribine tablets on lymphocyte reduction and repopulation dynamics in patients with relapsing multiple sclerosis. Mult Scler Relat Disord.

[160] Montalban X, Leist TP, Cohen BA, Moses H, Campbell J, Hicking C, Dangond F (2018). Cladribine tablets added to IFN-β in active relapsing MS: the ONWARD study. Neurol Neuroimmunol Neuroinflamm.

[161] Deeks ED (2018). Cladribine tablets: a review in relapsing MS. CNS Drugs.

[162] Neuhaus O, Kieseier BC, Hartung HP (2004). Mechanisms of mitoxantrone in multiple sclerosis--what is known?. J Neurol Sci.

[163] Scott LJ, Figgitt DP (2004). Mitoxantrone: a review of its use in multiple sclerosis. CNS Drugs.

[164] Hartung HP, Gonsette R, König N (2002). Mitoxantrone in progressive multiple sclerosis: a placebo-controlled, double-blind, randomised, multicentre trial. Lancet.

[165] Cocco E, Marchi P, Sardu C (2007). Mitoxantrone treatment in patients with early relapsing-remitting multiple sclerosis. Mult Scler.

[166] van de Wyngaert FA, Beguin C, D'Hooghe MB, Dooms G, Lissoir F, Carton H, Sindic CJ (2001). A double-blind clinical trial of mitoxantrone versus methylprednisolone in relapsing, secondary progressive multiple sclerosis. Acta Neurol Belg.

[167] Martinelli V, Radaelli M, Straffi L, Rodegher M, Comi G (2009). Mitoxantrone: benefits and risks in multiple sclerosis patients. Neurol Sci.

[168] Outteryck O (2016). Natalizumab in relapsing-remitting multiple sclerosis. Expert Rev Neurother.

[169] Havrdova E, Horakova D, Kovarova I (2015). Alemtuzumab in the treatment of multiple sclerosis: key clinical trial results and considerations for use. Ther Adv Neurol Disord.

[170] Syed YY (2018). Ocrelizumab: a review in multiple sclerosis. CNS Drugs.

[171] Hauser SL, Bar-Or A, Comi G (2017). Ocrelizumab versus Interferon Beta-1a in relapsing multiple sclerosis. N Engl J Med.

[172] Montalban X, Hauser SL, Kappos L (2017). Ocrelizumab versus placebo in primary progressive multiple sclerosis. N Engl J Med.

[173] Henze T, Rieckmann P, Toyka KV (2006). Symptomatic treatment of multiple sclerosis. Multiple Sclerosis Therapy Consensus Group (MSTCG) of the German Multiple Sclerosis Society. Eur Neurol.

[174] Toosy A, Ciccarelli O, Thompson A (2014). Symptomatic treatment and management of multiple sclerosis. Handb Clin Neurol.

[175] Abboud H, Hill E, Siddiqui J, Serra A, Walter B (2017). Neuromodulation in multiple sclerosis. Mult Scler.

[176] Samkoff LM, Goodman AD (2011). Symptomatic management in multiple sclerosis. Neurol Clin.

[177] Thompson AJ (2001). Symptomatic management and rehabilitation in multiple sclerosis. J Neurol Neurosurg Psychiatry.

